# SKELETAL MUSCLE OXIDATIVE CAPACITY AND LIFE COURSE PHYSICAL ACTIVITY IN PARTICIPANTS OF THE BLSA

**DOI:** 10.1093/geroni/igae098.3493

**Published:** 2024-12-31

**Authors:** Ann Zenobia Moore, Teresa Tian, Eleanor Simonsick, Luigi Ferrucci

**Affiliations:** National Institute on Aging, Baltimore, Maryland, United States; National Institute on Aging, Baltimore, Maryland, United States; National Institute on Aging, Baltimore City 0400 Baltimore City, Maryland, United States; National Institute on Aging, Baltimore, Maryland, United States

## Abstract

Physical activity has been associated with measures of mitochondrial quality and mitochondrial dysfunction and is connected to many aging processes. However, most studies relating physical activity to mitochondrial measures only capture physical activity over a limited period. The impact of engaging in physical activity across the life course on mitochondrial function has not been fully characterized. Here, in participants of the Baltimore Longitudinal Study of Aging we evaluate the association between a summary measure of life course physical activity (LCPA) and mitochondrial oxidative capacity, represented by postexercise phosphocreatine recovery rate (kPCr) in the quadriceps measured through 31P magnetic resonance spectroscopy. The study sample included 416 participants (43.5% male) with complete data. LCPA, derived from a physical activity history questionnaire by ranking participants’ sequences of reported activity intensity, starting with the current decade and ending with the response for physical activity during adolescence, was statistically significantly associated with kPCr (p <.01) in multivariable linear regression models adjusted for demographic characteristics and indicators of current physical activity. The positive association between LCPA and kPCr independent of current physical activity suggests that mitochondrial oxidative capacity in later life may have a cumulative dimension. Our observations demonstrate that even limited information on prior activity may be informative for understanding mitochondrial variability and highlight the importance of exercise across the life course.

